# Visceral Metastasis: A Prognostic Factor of Survival in Patients with Spinal Metastases

**DOI:** 10.1111/os.12657

**Published:** 2020-03-29

**Authors:** Deng‐xing Lun, Nai‐Wang Chen, Jiang‐tao Feng, Xion‐gang Yang, Zhao‐wan Xu, Feng Li, Yong‐cheng Hu

**Affiliations:** ^1^ Department of Spine Surgery Weifang People's Hospital Weifang China; ^2^ Graduate School of Tianjin Medical University Tianjin China; ^3^ Department of Bone Oncology Tianjin Hospital Tianjin China

**Keywords:** Meta‐analysis, Metastatic spinal cord compression, Prognosis, Survival, Visceral metastasis

## Abstract

**Objective:**

To characterize the visceral metastasis as a predictive tool for the survival of patients with spinal metastases through an exploratory meta‐analysis.

**Methods:**

Two investigators independently searched PubMed and Embase databases for eligible studies from 2000–2016. The effect estimates for the hazard ratio (HR) or risk ratio (RR) and 95% confidence interval (CI) were collected and pooled with a random‐ or fixed‐effect model.

**Results:**

In total, 18 eligible studies were retrieved with 5468 participants from nine countries. The overall pooled effect size for HR and RR was 1.50 and 3.79, respectively, which was proved to be statistically significant. In the subgroup of prostate cancer (PCa) and non‐small cell lung cancer (NSCLC), statistical significance and marginal statistical significance was presented for the pooled HR (HR = 1.76, 95% CI 1.35–2.29) and (RR = 1.56, 95% CI 0.99–2.48), respectively. However, in the subgroup of thyroid cancer, breast cancer, and renal cancer, statistical significance was not achieved (HR = 1.17, 95% CI 0.75–1.83, *Z* = 0.70, *P* = 0.486). The results did not show any evidence of publication bias.

**Conclusions:**

This study demonstrated that visceral metastasis was a significant prognostic factor in patients with spinal metastases as a whole. Interestingly, the onset of visceral metastases differentially impacted the survival in different primary tumors. Therefore, the prognostic value of visceral metastasis might be related to the type of primary tumor.

## Introduction

The prevalence of symptomatic spinal metastasis has increased due to improved treatments and prolonged survival in cancer patients. Approximate 70% of the patients with cancer can develop spinal metastases^1^, ^2^, among whom 20% are usually suffering from neurological deficits^3^, ^4^. For metastatic spinal cord compression, approximately 10% of such patients would choose to undergo surgical decompression with/without stabilization^1^, ^2^, ^4^, ^5^, ^6^, ^7^, ^8^, which can restore neurological function and improve quality of life. However, the mechanism to identify the patients who may benefit maximally from surgical treatment is not yet clear. Thus, decompressive surgery is not indicated for patients with a severely limited lifespan for only a few weeks^9^, while supportive care or radiotherapy is appropriate^10^. Therefore, life expectancy drives treatment regimens for spinal metastasis^9^, ^10^, ^11^, ^12^.

Currently, several prognostic scoring systems have been proposed to predict the life expectancy in patients with metastatic spinal cord compression (MSCC)^13^, ^14^, ^15^, ^16^, ^17^, ^18^, primarily including several parameters such as general condition, the extent of extraspinal bone metastases, the extent of spinal metastases, visceral metastases, primary tumor, the severity of spinal cord palsy, and pathological fracture. Of these, visceral metastasis is one of the most valuable prognostic factors in several previous studies^13^, ^15^.

Although Tomita *et al*.^13^, Tokuhashi *et al*.^14^, ^15^, Sioutos *et al*.^16^, North *et al*.^17^, van der Linden *et al*.^18^, Bauer and Wedin^19^, and Leithner *et al*.^20^ regarded visceral metastasis as a critical component of the scoring systems, nearly half of the studies reported controversial results of its prognostic effect on patients. Ju *et al*.^21^ demonstrated that visceral metastasis did not associate significantly with the survival in patients with MSCC from prostate cancer. Moreover, Zadnik *et al*.^22^ reported that the difference in survival was not significant between patients with visceral metastases (median survival, 25.9 months) as compared to those without visceral metastases (median survival, 28.1 months). In addition, visceral metastasis is considered to be in the terminal stage of their disease, thereby necessitating only palliative treatments. However, Walcott *et al*.^23^ concluded that the existence of a progressive systemic disease should not be a contradiction to aggressive surgery. Therefore, visceral metastasis was a controversial prognostic factor in patients with spinal metastases.

The current meta‐analysis is performed with the goal of identifying and quantifying the role of visceral metastasis in predicting the survival time in patients with spinal metastases.

## Methods

### *Collection of Published Literature*

We performed a systematic search in PubMed and Embase databases for eligible publications. The following terms were used: “Visceral metastasis,” “Prognosis,” “Survival,” and “Metastatic spinal cord compression.”

Searching strategies for PubMed and Embase were applied as below:

(i) PubMed

#1 Visceral metastasis

#2 lung

#3 prostate

#4 kidney

#5 thyroid

#6 breast

#7 #1#2#3#4#5#6

#8 spinal metastases

#9 #7 AND #8

((((((((lung) OR prostate) OR kidney) OR thyroid) OR breast)) OR Visceral metastasis)) AND spinal metastases

(ii) Embase

#1 ‘Visceral metastasis'/exp OR ‘lung':ab,ti OR ‘prostate':ab,ti OR ‘kidney':ab,ti OR ‘thyroid':ab,ti OR ‘breast':ab,ti

#2 ‘spinal metastases’/exp

#3 #1 AND #2

### *Inclusion and Exclusion Criteria for Studies*

Studies were included if the following criteria were fulfilled: (i) patients were diagnosed with spinal metastases; (ii) patients who received surgery or radiotherapy; (iii) survival outcomes and prognostic factors were analyzed; and (iv) in order to avoid the impact of targeted therapeutic drugs on the results, only published papers in the English language between January 2000 and December 2016 were searched. All the potentially relevant articles were reviewed and extracted independently by two investigators; the disagreements were resolved by discussion, and the consensus was finally reached.

The exclusion criteria were as follows: (i) the nature of the study was a systematic review, basic research, letter to editors, sensitive analysis, or diagnostic study; (ii) there are <10 participants included; (iii) studies with repeated patients' cohorts; and (iv) duplicated studies.

### *Data Extraction and Quality Assessment*

Two reviewers extracted the data from eligible articles independently, discussed discrepancies, and reached conformity with respect to all parameters. The indispensable information extracted from all primary studies included baseline characteristics (title, author, year of publication, country, period of the study, and study design), participants' characteristics (age, percentage of males, number of involved patients, number of patients with MSCC, and primary tumor type), effect sizes of hazard ratio (HR) or risk ratio (RR) coupled with respective 95% confidence interval (95% CI). In addition, if HR or 95% CI was not estimated directly, related raw data, such as the survival rates of specific time points and Kaplan–Meier survival curves, were collected by Get Data Graph Digitizer software (version 2.25, http://getdata-graph-digitizer.com) that was calculated rather indirectly.

The Excel spreadsheet^24^ is also used in the calculation. Diversities on the obtained information were deduced, and disagreements were discussed in person. In the event that several cohorts were studied among the similar population, the newest or most impeccable survey was applied.

The Newcastle–Ottawa Scale (NOS)^25^ was used to assess the quality of the method and risk of bias by the two researchers independently as described previously. The scale employed a 9‐star system that assessed three domains: patient selection, comparability of the study groups, and ascertainment of the study outcome. A score of 9 stars indicated low risk of bias, whereas 7–8 indicated medium risk of bias, and a score ≤6 indicated a high risk of bias.

### *Data Synthesis and Analysis*

Data, extracted into the Microsoft Excel spreadsheet, were pooled by an exploratory time‐to‐event meta‐analysis. All recorded HRs or RRs were combined with 95% CI (including statistically significant or non‐significant) from eligible literature, incorporating the HRs re‐calculated from raw data or Kaplan–Meier curves obtained from primary studies that were synthesized narratively. The pooled estimate for HR or RR and 95% CI of visceral metastasis was deduced using the random‐effect or fixed‐effect model^26^.

The heterogeneity assumption was verified by Q‐test. A significant Q‐test value (*P* < 0.10) indicated heterogeneity across studies, following which, the random‐effect model would be selected. On the other hand, the fixed‐effect model would be selected. The significance of the pooled effect was determined by the *Z*‐test (*P* < 0.05 was considered to be statistically significant). One‐way sensitivity analyses were performed to assess the stability of the results, termed as a single study in the meta‐analysis that was deleted each time to reflect the influence of the individual dataset on the pooled effect size. An estimate of potential publication bias was carried out by the funnel plot. An asymmetric plot suggested a potential publication bias. The asymmetry of the funnel plot was assessed by Egger's test. The significance of the intercept was determined by the *t*‐test, as suggested by the Egger's test (*P* < 0.10 was considered as statistically significant publication bias). Subgroup analyses were performed according to the participants, primary tumor histology, and effect estimates of HR or RR in each study. All statistical tests were performed using Stata (version 13.0, StataCorp LLC, College Station, Texas, USA) with two‐sided *P*‐values.

## Results

### *Search Result and Data Extraction*

**Figure 1:**
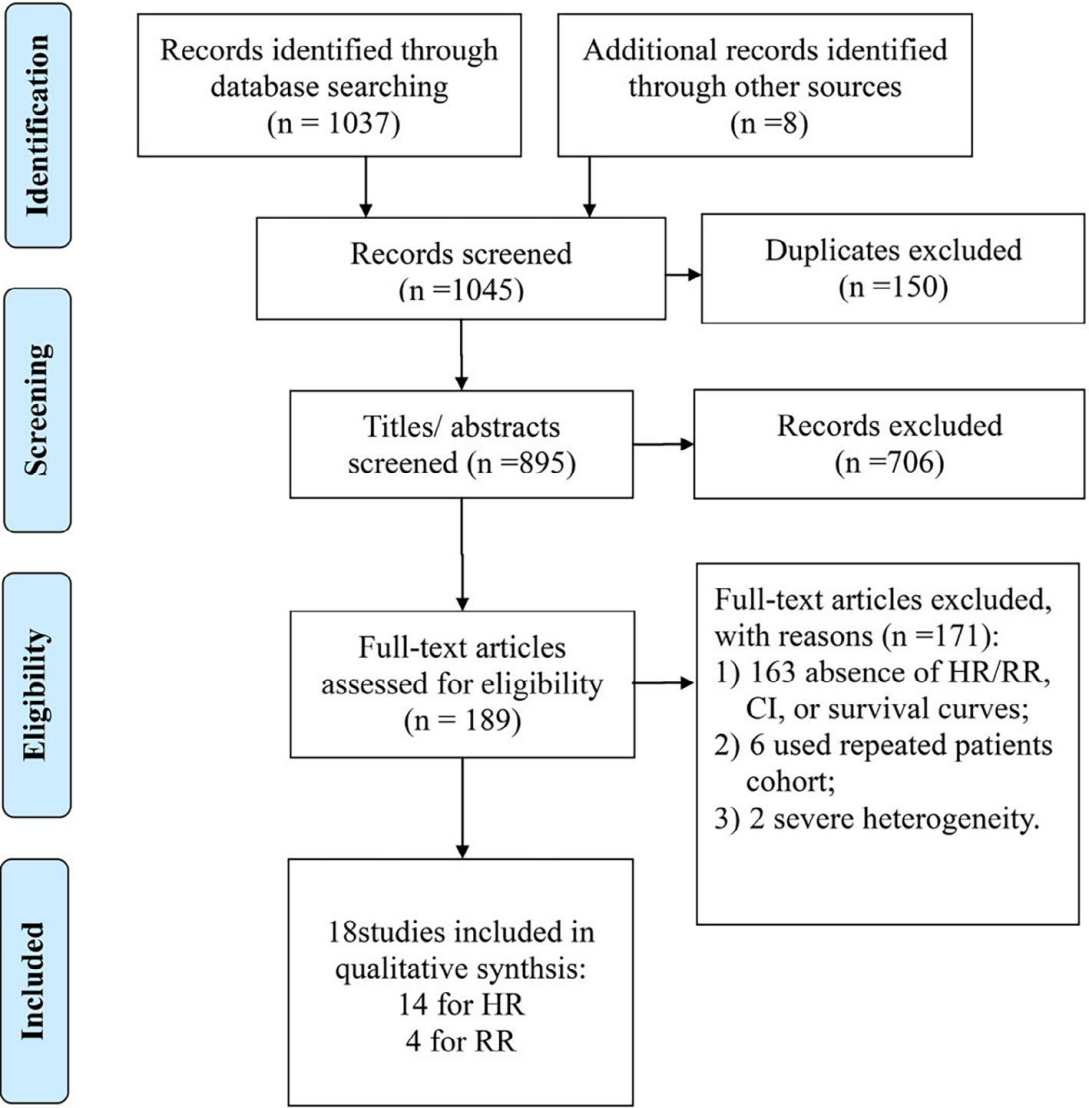
Schematic representation of studies' identification and selection.

The initial search retrieved 1045 articles. After 150 duplicates were excluded, 895 articles remained. Then, after scrutinizing the titles and abstracts, 706 studies were excluded. A further 163 studies were excluded due to the absence of HR, 95% CI, or survival curves. 1/163 study^27^ did not present an effect size for HR in multivariate analysis although it significantly originated from visceral metastasis as assessed by univariate analysis and was included in multivariate Cox proportional hazard model. Six studies with the selection of repeated patients were excluded^28^, ^29^, ^30^, ^31^, ^32^, ^33^. Another two studies (HR = 199.239, 95% CI 2.615–15180.426 and HR = 5.55, 95% CI 2.39–12.89, respectively)^34^, ^35^ were excluded due to severe heterogeneity with other studies according to sensitivity analysis. Finally, 18 studies^18^, ^20^, ^21^, ^22^, ^36^, ^37^, ^38^, ^39^, ^40^, ^41^, ^42^, ^43^, ^44^, ^45^, ^46^, ^47^, ^48^, ^49^ containing 5468 patients fulfilled the inclusion criteria, and hence were enrolled in the meta‐analysis that consisted of 14 studies with an effect size for HR and four for RR. The schematic representation of the literature search was shown in Fig. 1.

### *Characteristics of Included Studies*

A summary of individual studies was listed in Table 1. In 16 studies with age reported, there were 1883 males and 1535 females, with a mean age of 61.4 years (from 27 to 91 years). The histology of primary tumors varied among 18 studies, with six non‐specified containing 4148 participants, three non‐small cell lung cancer (NSCLC) with 470 participants, four prostate cancer with 646 participants, two thyroid cancer with 53 patients, two breast cancer with 130 patients, and one renal cell cancer containing 646 participants. Three thousand seven hundred and ninety‐two patients in 11 studies presented with progressive neurological deterioration by metastatic spinal cord compression. Of those, 2945 patients in four studies^18^, ^45^, ^47^, ^48^ were treated with radiotherapy alone, 733 patients in five studies^21^, ^36^, ^37^, ^40^, ^49^ received surgery plus adjuvant therapy, 64 patients in one study by Lei *et al*.^38^ received surgery alone, 50 patients [40] were treated with surgery and other adjuvant therapy. The axial pain was the main clinical symptom in seven studies with 2290 patients. Of those,1493 patients in three studies^20^, ^43^, ^46^ received surgery plus adjuvant therapy, 140 patients in three studies^41^, ^42^, ^44^ with surgery alone, 43 ones^22^ with surgery and other adjuvant therapy. Studies were conducted in different countries: five in the USA, three in Germany, three in China, three in the Netherlands, and one each in Japan, Sweden, Austria, South Korea, and Dutch. With respect to the delimitation, 17 were retrospective and only one was a semi‐retrospective cohort with prospective manner for assimilation of the information. All studies were high quality with an average score of 7.9 ± 0.9 stars; only one study had a score of 6.0 stars.

**Table 1:** Summary of included studies

Author	Year	Country	Study period	Study design	Patients involved	Patients with MSCC	Male%	Primary tumor	Age (years)	Overall survival	Therapeutic modality	NOS
Van der Linden *et al*.^18^	2005	Dutch	Mar. 1996–Sept. 1998	Retrospective cohort	342	12	53	NI	Mean (min‐max): 66 (34–90)	Mean: 11 months; median: 7 months	RT alone	8
Leithner *et al*.^20^	2008	Austria	Jan. 1998–Sept. 2006	Prospective + retrospective	69	NS	54	NI	Mean (min‐max): 60 (30–79)	OS% (12 months): 28 median: 14 months	SUR+RT, *et al*.	7
Arrigo *et al*.^36^	2011	USA	1999–2009	Retrospective cohort	200	172	61	NI	Mean (min‐max): 58.9 (19–89)	OS% (1 year, 3 year, 5 year): 38.3, 21.1, 9.41	SUR+RT, *et al*.	9
Chong *et al*.^37^	2012	Korea	Mar. 2002–Jun. 2010	Retrospective cohort	105	105	69	NI	Mean (min‐max): 58.3 (30,87)	Median: 6 months; OS% (1 year, 2 year): 34, 14	SUR+RT, *et al*.	8
Rades *et al*.^45^	2013	Germany	1992–2011	Retrospective cohort	2029	2029	NS	NI	NS	OS% (2months): 80	RT alone	8
Bollen *et al*.^46^	2014	Netherlands	Jan. 2001–Dec. 2010	Retrospective cohort	1403	NS	52	NI	Mean: 64.8 (±12.5)	Median: 4.8 months; OS% (6 weeks, 2 year): 77, 17	SUR/RT/(SUR+RT)	9
Rades *et al*.^47^	2012	Germany	1992–2010	Retrospective cohort	356	356	74	NSCLC	Median:64	OS% (6 months, 12 months): 27.7, 13.5	RT alone	9
Lei *et al*.^38^	2015	China	May. 2005–May. 2015	Retrospective cohort	64	64	66	NSCLC	Median:57	Median: 6.3 months； OS% (6 months, 12 months): 52. 6, 23	SUR, *et al*.	9
Chen *et al*.^39^	2015	China	Nov. 2000‐ Mar. 2010	Retrospective cohort	50	50	68	NSCLC	Mean (min‐max): 61.6 (20–87)	Median: 7.5 months	SUR & other adjuvant therapy	8
Ju *et al*.^21^	2013	USA	Jun. 2002‐ Aug. 2011	Retrospective cohort	27	27	100	PCa	Median (min‐max): 65 (46,82)	OS% (1 m, 3 months, 6 months, 9 months, 12 months): 96, 81, 70, 62, 40	SUR+RT, *et al*.	8
Rades *et al*.^48^	2012	Germany	1992–2010	Retrospective cohort	218	218	100	PCa	NS	OS% (6 months,12 months): 62.2， 48.9	RT alone	7
Crnalic *et al*.^49^	2012	Sweden	Sept. 2003–Sept. 2010	Retrospective cohort	68	68	100	PCa	Median:71	OS% (3 months, 6 months, 12 months, 24 months): 64, 45, 30, 8	SUR+RT, *et al*.	7
Drzymalski *et al*.^40^	2010	USA	Jun. 1990–Apr. 2009	Retrospective cohort	333	77	100	PCa	Median (min‐max): 68 (43,90)	Median: 24 months OS% (12 months): 73	SUR/RT, *et al*.	8
Bakker *et al*.^43^	2014	Netherlands	Jan. 2006–Jul. 2013	Retrospective cohort	21	NS	NS	RCC	NS	Median (min‐max): 25 months (11 months, 75 months)	SUR+RT, *et al*.	6
Kato *et al*. 41	2016	Japan	1984–2011	Retrospective cohort	32	NS	22	TCa	Mean: 60.6	Median:6.4 year OS% (5 year, 10 year): 71%, 31%	SUR, *et al*.	7
Liang *et al*.^42^	2014	China	1999–2013	Retrospective cohort	21	NS	24	TCa	NS	NS	SUR, *et al*.	7
Sciubba *et al*.^44^	2007	USA	Jun. 1993–Jun. 2001	Retrospective cohort	87	NS	0	BCa	Median (min‐max): 53 (35,84)	OS% (1 year, 2 year, 3 year, 4 year, 5 year): 62, 44, 33, 27, 24	SUR, *et al*.	9
Zadnik *et al*.^22^	2004	USA	Jun. 2002–Aug. 2011	Retrospective cohort	43	NS	0	BCa	Median (min‐max): 56 (27,91)	Median:26.8 months	SUR & other adjuvant therapy	8

BCa, breast cancer; MSCC, metastatic spinal cord compression; NI, not identified primary tumor type; NOS, Newcastle–Ottawa Scale; NS, not specified; NSCLC, non‐small cell lung cancer; OS%, percentage of overall survival; PCa, prostate cancer; RCC, renal cell cancer; RT, radiotherapy; SUR, surgery; TCa, thyroid cancer.

### *Qualitative Summary and Data Synthesis*

Among the 18 studies included, only eight studies reported statistically significant results. Moreover, 14 studies presented the effect sizes for HR. Among these studies, all cohorts were involved with surgical procedures except one, wherein only radiotherapy was administered^18^. In this study, the HR was 1.67 (CI 95% 1.25–2.50), with a significant result (*P* < 0.001). Additionally, four studies that presented effect sizes for RR were treated with radiotherapy alone.

For the HR group (Fig. 1), all the effect estimates for HR were synthesized narratively by a subgroup meta‐analysis based on the primary tumor histology using the fixed‐effect model. The overall pooled effect size for HR was 1.50 (95% CI 1.36–1.66), which was substantiated as statistically significant. However, the subgroups of the thyroid, breast, and renal cancers did not achieve statistical significance (HR = 1.17, 95% CI 0.75–1.83, *Z* = 0.70, *P* = 0.486). In NSCLC subgroup, a marginal statistical significance was presented for the pooled HR (HR = 1.56, 95% CI 0.99–2.48, *Z* = 1.90, *P* = 0.058), whereas in the subgroup of prostate cancer, statistical significance was presented for the pooled HR (HR = 1.76, 95% CI 1.35–2.29).

**Figure 2:**
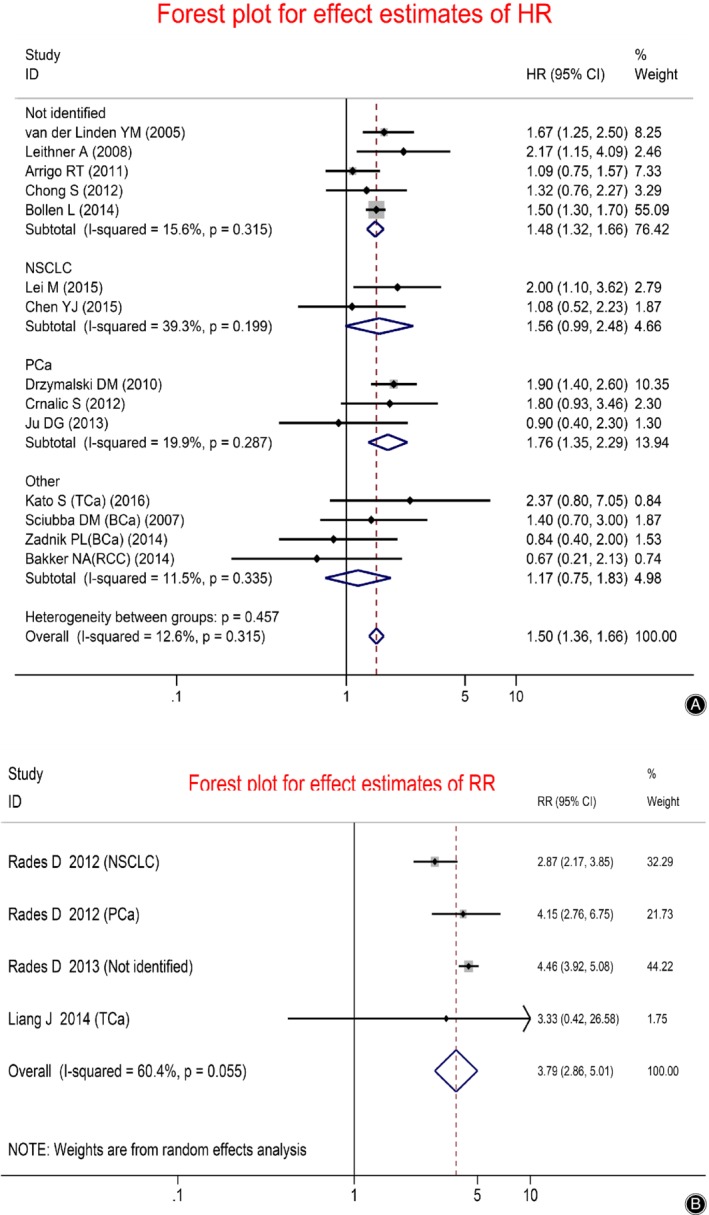
Forest plot presenting the effect estimates of survival in patients with spinal metastases: (A) The effect sizes for HR between patients with and without visceral metastases; (B) The effect sizes for RR between patients with and without visceral metastases.

For the RR group including three radiotherapy cohorts and one surgery cohort (Fig. 2), the effect estimates were pooled by the random‐effect meta‐analysis. The pooled effect size for RR was 3.79 (95% CI 2.86–5.01) and *I* ^*2*^ = 60.4%, which was proved to be significant by the *Z*‐test (*Z* = 9.33, *P* < 0.001).

### *Publication Bias*

**Figure 3:**
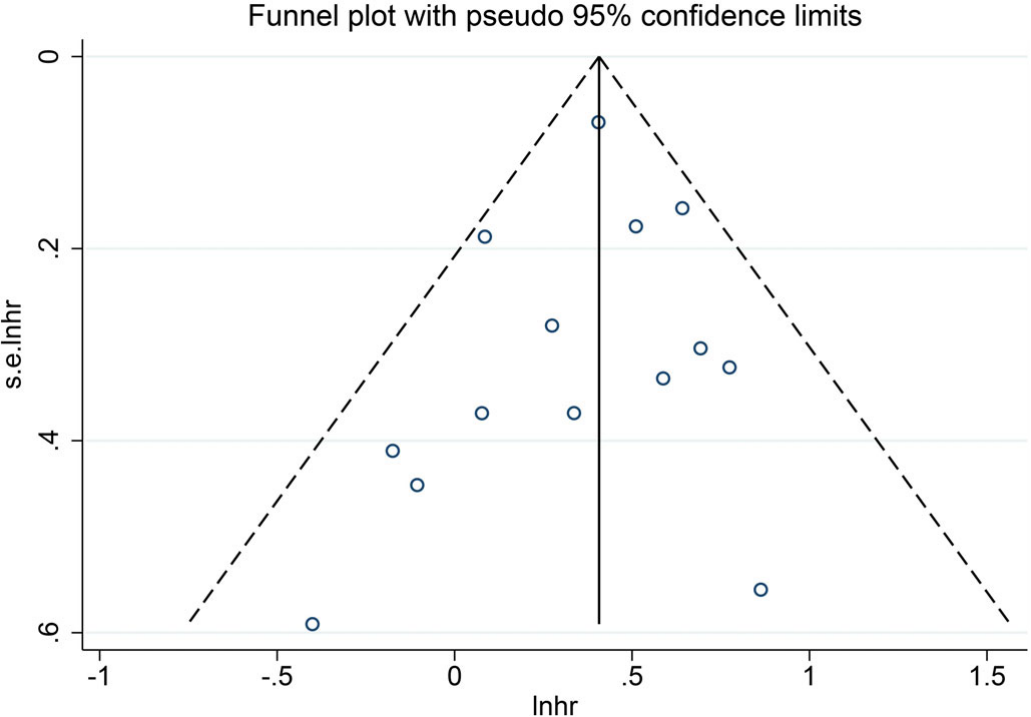
Funnel plot presenting the publication conditions of 14 studies included in the forest plot A. A relative symmetry was presented visually.

**Figure 4:**
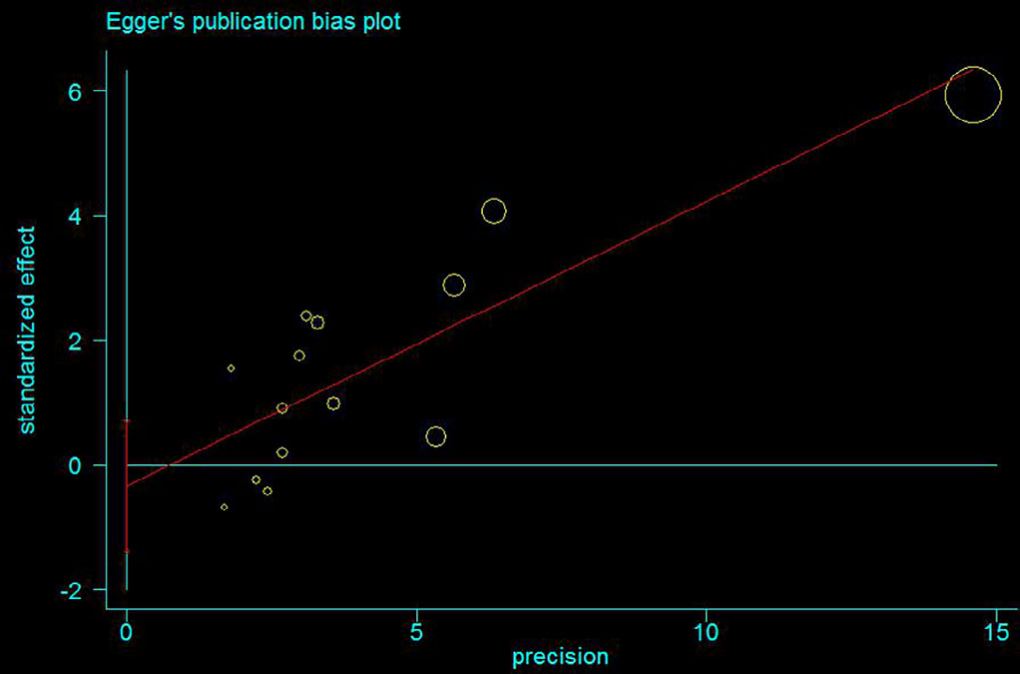
Egger's publication bias plot presenting the risk of bias across 14 studies involved in the forest plot A. The *P*‐value = 0.505 was considered statistically insignificant, and thus, the publication bias was not existent.

**Figure 5:**
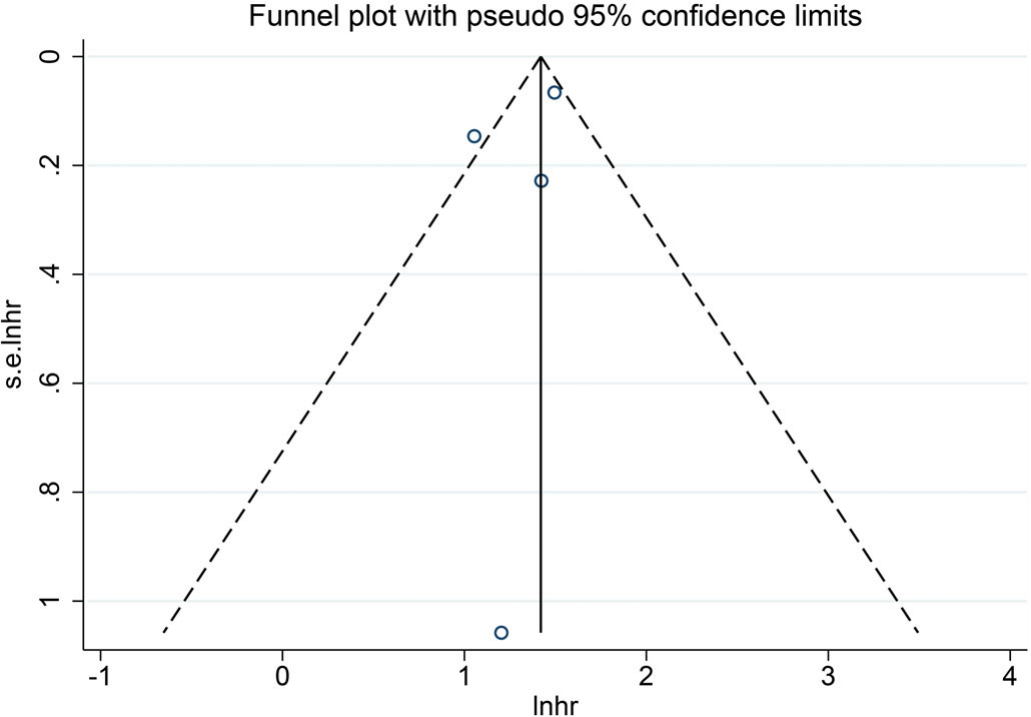
Funnel plot presenting the publication conditions of four studies included in the forest plot B. A favorable symmetry was not presented visually, and thus, the publication bias might be present.

**Figure 6:**
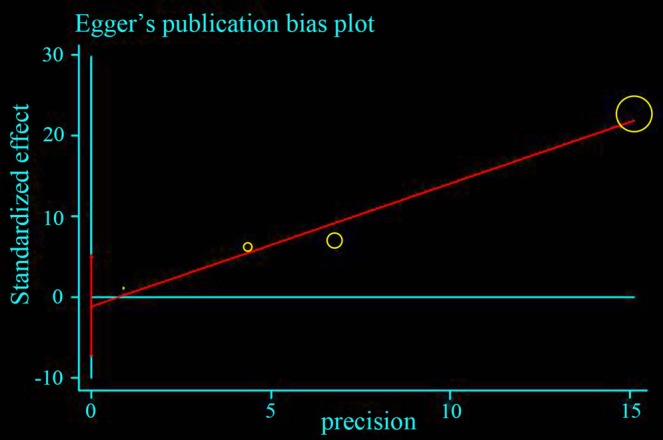
Egger's publication bias plot presenting the risk of bias across four studies included in the forest plot B. The *P*‐value = 0.267 was considered non‐significant, and thus, the publication bias was not statistically significant.

Funnel plot and Egger's test were performed to assess the publication bias (Figs 3, 4, 5, 6). The shape of the funnel plots did not reveal any evidence of obvious asymmetry in all the genetic models, and Egger's test provided statistical evidence. The results did not show any evidence of publication bias.

## Discussion

To the best of our knowledge, this study is the first meta‐analysis for identifying the role of visceral metastasis in predicting the overall survival in patients with spinal metastases, providing useful information for physicians and surgeons. This study can clarify the current controversies in the field and render visceral metastasis as a remarkable prognostic factor for different pathological sub‐types. The study fully complied with a standard protocol, and all procedures were conducted by two physicians individually.

However, whether visceral metastasis is a prognostic factor for survival in patients with spinal metastases is controversial in current literature. Visceral metastasis was regarded as a major prognostic factor in various scoring systems^13^, ^14^, ^15^, ^16^, ^17^, ^18^, ^19^, ^20^ as it indicates aggressive tumor and shortens the life span of patients considerably. However, several studies^21^, ^22^ were opposed to the implementation of visceral metastasis as a significant prognostic factor based on their cohorts, which might be attributed to a greater degree of malignancy in spinal metastases than visceral metastases in specific tumors, such as lung cancer. Leithner *et al*.^20^ found that primary tumor and visceral metastases were the only significant parameters, according to multivariate analysis, in which all the seven parameters were assessed: general condition, extent of extraspinal bone metastases, extent of spinal metastases, visceral metastases, primary tumor, severity of spinal cord palsy, and pathological fracture. Tabouret *et al*.^50^ reported that multiple systemic metastases were not significantly predictive of survival, although a comparative analysis among the long‐term survivors and the other patients with overall survival <2 years exhibited significant differences. Arrigo *et al*.^36^ reported that visceral metastasis was not a significant predictor of mortality as evaluated from 200 surgically‐treated spinal metastasis patients. Chong *et al*.^37^ investigated preoperative prognostic factors of 108 patients, and the multivariate Cox proportional hazard model revealed that although the median survival of the patients with and without visceral metastases at the time of surgery was 4 and 11 months, respectively, it was not an independent prognostic factor. Lun *et al*.^51^ also determined that visceral metastases do not appear to predict the prognosis of patients with MSCC for some primary tumors. The current study focused on evidence‐based medicine, demonstrating that visceral metastasis is a statistically significant prognostic factor for overall survival after complete treatment, with pooled overall HR 1.50 (95% CI 1.36–1.66) as compared to patients with and without visceral metastasis.^52^

In the case of non‐small cell lung cancer, whether visceral metastasis affected the survival rate of patients, Lei *et al*.^38^ reported that visceral metastases significantly affected the survival as assessed by multivariate analysis, and Chen *et al*.^39^ reported a contradictory result, that visceral metastasis did not significantly associate with the survival in NSCLC patients with spinal metastases who underwent spinal surgery. In this study, the pooled effect estimate was 1.56 (95% CI 0.99–2.48) (*Z* = 1.90, *P* = 0.058), which showed that the relationship between visceral metastasis and survival prognosis was marginally significant. Because the effective valve of negative results would not be reported in the excluded studies,^53^ the prognostic impact of visceral metastases on survival is suspected; thus, further study is essential.

For patients with MSCC from prostate cancer, visceral metastasis did affect the survival rate of patients. Drzymalski *et al*.^40^ found that the presence of additional metastasis during the diagnosis of spinal metastasis was independently associated with a short overall survival. Crnalic *et al*.^35^ reported that visceral metastasis had a detrimental effect on the survival for prostate cancer, and found that the median survival in patients with visceral metastases was only 4 months as compared to 10 months in patients without visceral metastases. On the contrary, Ju *et al*.^21^ demonstrated that visceral metastases had no significant association with survival in patients with MSCC from prostate cancer. However, the pooled effect estimate was 1.76 (95% CI 1.35–2.29), which represented optimal correlations between visceral metastases and survival prognosis.

For patients with spinal metastases from thyroid cancer, no correlation was established between visceral metastasis and prognosis post‐surgery. Only two articles included in the meta‐analysis showed no statistical significance. Kato *et al*.^41^ reported that the presence of lung metastases was not associated with survival because lung metastases respond to radioiodine treatment as compared to the metastases of other organs. Sellin *et al*.^27^ reported visceral metastases did not affect patients' prognosis as assessed by multivariate analysis, although univariate analysis demonstrated a significant association with poor overall survival. Jiang *et al*.^42^ found that no significant effects on postoperative recurrence or survival were observed in the absence and presence of visceral metastasis.

According to the Tomita score, breast and kidney cancer were speculated to grow slowly and moderately, respectively. In the original and modified Baur score, the breast and kidney lesions could be compared to visceral metastasis and regarded as independent prognostic factors. However, Bakker *et al*.^43^ found that visceral metastasis was not associated significantly with survival in patients with renal cell carcinoma. Walcott *et al*.^23^ found that the concomitant presence of visceral lesions or multi‐focal bony disease did not exert a prognostic significance in patients with breast cancer. Although Han *et al*.^54^ demonstrated that visceral metastases significantly affected the patients' survival in univariate analysis, and the multivariate Cox regression model showed that it was not a prognostic factor in patients with renal cancer. In addition, Zadnik *et al*.^22^ examined the relationship of visceral metastases to survival in patients with MSCC from breast cancer and found that the median survival in the group with no visceral metastases was 25.9 months as compared to 28.1 months in the group with visceral metastases. The difference in the survival was not significant between groups as assessed by Mantel–Cox testing. In another study by Sciubba *et al*.^44^, the median survival of patients without visceral metastases was 28.0 months as compared to 17.4 months in the cases with visceral metastases. However, the result of multivariate analysis was similar to that by Zadnik *et al*.^22^ without a statistical difference. The current study showed that visceral metastasis was not a vital prognostic factor in breast and renal cancers, and the pooled effect estimate was only 1.11 (95% CI 0.65–1.91) and 0.67 (95% CI 0.21–2.13), respectively.

### *Limitations*

Notably, our study has several limitations. First, some studies did not present an effect on the size in the multivariate analysis of overall survival; however, the Kaplan–Meier curves or survival rates at specific time points affected the estimate of HRs that was deduced from the raw data. Although these features exerted an inevitable bias on the actual results, it has become a widely accepted method when actual data is unavailable from primary literature as the estimates were similar to real results. Moreover, these data were indispensable as the reporting bias was subsistent; these studies did not present results that were insignificant in multivariate analysis and could be reflected as results obtained from raw data that were not statistically significant. Additionally, studies included in the meta‐analysis were high‐quality observational cohort designs. The absence of any randomized controlled trial (RCT) might be attributed to the few RCTs carried out to date. In addition, a majority of the studies were high‐quality according to NOS. Finally, only a few studies reported the prognostic effect of visceral metastasis in patients with spinal metastases from primary tumors such as renal cancer and breast cancer. However, we obtained valuable results from a meta‐analysis from the evidence‐based medical methods.

### *Conclusions*

The current study suggested that the occurrence of visceral metastases has a strong negative impact on survival and should be considered while choosing a precision treatment. Interestingly, the onset of visceral metastases exhibited various impacts on survival in different primary tumors. However, visceral metastasis in thyroid cancer, breast cancer, and renal cancer cannot yet be confirmed as a significant prognostic factor for survival, thereby necessitating further studies. Thus, large prospective trials are required to better define the prognostic value of visceral metastasis in a patient with different tumors.
